# Association of congenital anomalies with fracture of spine, trunk, and upper and lower limbs among young people: A population-based matched cohort study in Taiwan

**DOI:** 10.1097/MD.0000000000007980

**Published:** 2017-09-08

**Authors:** Chia-Man Ma, Lih-Hwa Lin, Hsing-Yi Chang, Pei-Chi Chou, Po-Chi Liao, Huey-Yi Chen, Kee-Ming Man, Jen-Huai Chiang, Yin-Jen Chang, Ming-Yen Tsai, Wen-Chi Chen, Yung-Hsiang Chen

**Affiliations:** a Department of Dermatology, Department of Urology, Taichung Veterans General Hospital, Taichung; b Division of Chinese Medicine, An Nan Hospital, China Medical University, Tainan; c Graduate Institute of Chinese Medicine, School of Chinese Medicine, Graduate Institute of Integrated Medicine, Research Center for Chinese Medicine & Acupuncture, College of Medicine, China Medical University, Taichung; d Institute of Population Health Sciences, National Health Research Institutes, Miaoli; e Departments of Chinese Medicine, Obstetrics and Gynecology, Anesthesiology, Urology, and Medical Research, Management Office for Health Data, China Medical University Hospital, Taichung; f Department of Medicinal Botanicals and Health Applications, Da-Yeh University, Changhua; g Department of Chinese Medicine, Kaohsiung Chang Gung Memorial Hospital and Chang Gung University College of Medicine, Kaohsiung; h Department of Psychology, College of Medical and Health Science, Asia University, Taichung , Taiwan.

**Keywords:** congenital anomalies, kidney, nationwide cohort study, traditional Chinese medicine theory

## Abstract

According to the Traditional Chinese Medicine (TCM) theory, congenital anomalies are caused by kidney malfunctions, which decreased the bone quality, and may eventually result in bone fractures. This retrospective cohort study investigated the relationship between congenital anomalies and fracture of spine, trunk, and upper and lower limbs in young people. We utilized data from the National Health Insurance Research Database of Taiwan. This study included patients with congenital anomalies (International Classification of Diseases/ICD-9 code: 740–759) and a comparison group of patients without congenital anomalies. Cases evaluated were fracture of spine and trunk (ICD-9 codes: 805–809), fracture of upper limbs (ICD-9 codes: 810–819), and fracture of lower limbs (ICD-9 codes: 820–829). Our study shows that in comparison to the control group, patients with congenital anomalies are 1.11 times more likely to develop fractures. This is the first documented research study that supports the TCM theory that “the Kidney governs the bones, and healthy bones give the body stabilization and prevent fracture.”

## 1 Introduction

Congenital anomalies, also known as congenital diseases, deformities, birth defects, or abnormalities, are conditions existing at or before birth.^[1,2]^ According to the 2002 birth registries, congenital anomalies occurred in 0.7% of all births in Taiwan.^[3]^ The severity of birth defects varies. Some congenital anomalies are minor and correctable, whereas others cause severe mental and physical disabilities, and some may be fatal.^[3]^ Congenital anomalies are medical and public health issues that can result in physical and psychological suffering and financial burden. It is well known that congenital anomalies are related to maternal age. However, the relationship between congenital anomalies and fractures of the spine, trunk, and upper and lower limbs, in young people is not well documented.

A basic concept of Traditional Chinese Medicine (TCM) states there are 5 important endoorgans that govern other organs or tissues.^[4]^ The 5 endoorgans are the liver, heart, spleen, lung, and kidney. This concept states that the Kidney governs the quality of bones, which can be correlated with fracture. According to the TCM theory, congenital anomalies are caused by kidney malfunctions, which lower bone quality, eventually causing fracture. Following this principle, we investigated the correlation between congenital anomalies and fractures. This investigation was based on a nationwide, population-based cohort study in Taiwan.

## 2 Materials and methods

### 2.1 Database

This is a retrospective cohort study and was based on collected data from the National Health Insurance Research Database (NHIRD). These data were obtained from health care data comprising about 96% of all medical records in Taiwan since 1996. Medical services provided by the NHI program included both Western and TCM concerning out-patient care, in-patient care, physical therapies, dental services, prescription drugs, medical institutions, and registration files with scrambled identifications.^[5]^

We compiled data files for children (age < 18 years) from the NHI program, which were established and maintained by the National Health Research Institutes (NHIR). The children data set consisted of a randomly selected sample of half of all children in Taiwan. The cohort period is from year 2000 to 2006 and a follow-up monitoring until the end of 2008. The diagnoses codes used were from the International Classification of Diseases 9th Revision of the Clinical Modification (ICD- 9-CM) in the database. This study was approved by the Institutional Review Board of China Medical University Hospital (CMUH-104-REC2-115 and CRREC-103-048).

The study population was newly diagnosed with congenital anomalies patients (ICD-9 code: 740–759) from year 2000 to 2006 as the case cohort. Different congenital anomalies classifications are obtained, that is, nervous system, eye and face, cardiovascular, digestive, urogenital, musculoskeletal, respiratory, and chromosomal anomalies. The comparison cohort was patients with noncongenital anomalies (control group). We matched the control group with the case cohort, which is 4 times bigger in number, according to their ages, genders, and index years. The ages were between 0 and 18 years old in both groups. The cases were diagnosed at least twice as fracture of the spine and trunk, fracture of the upper limb, and fracture of the lower limb (ICD-9 code: 805–809, 810–819, and 820–829 separately) between the years 2000 and 2008. The patients were monitored until the end of the follow-up study in 2008/12/31.

Patients with fracture previously diagnosed with congenital anomalies were excluded. We also excluded patients who suffered the same fracture because of trauma (E-code).

### 2.2 Statistical analyses

A Cox model was used to estimate hazard ratio (HR) and 95% CI (confidence intervals) for the characteristics associated with gender and congenital anomalies. All analyses were performed using the SAS statistical package (SAS System for Windows, Version 9.4) with statistics adapting the significance level at 0.05.

## 3 Results

We identified a total of 87,727 patients coded with congenital anomalies and 350,908 coded patients without congenital anomalies. The patients coded without congenital anomalies, 4 times the number of patients with congenital anomalies, served as the control group. The demographic data are listed in Table 1.

**Table 1:**
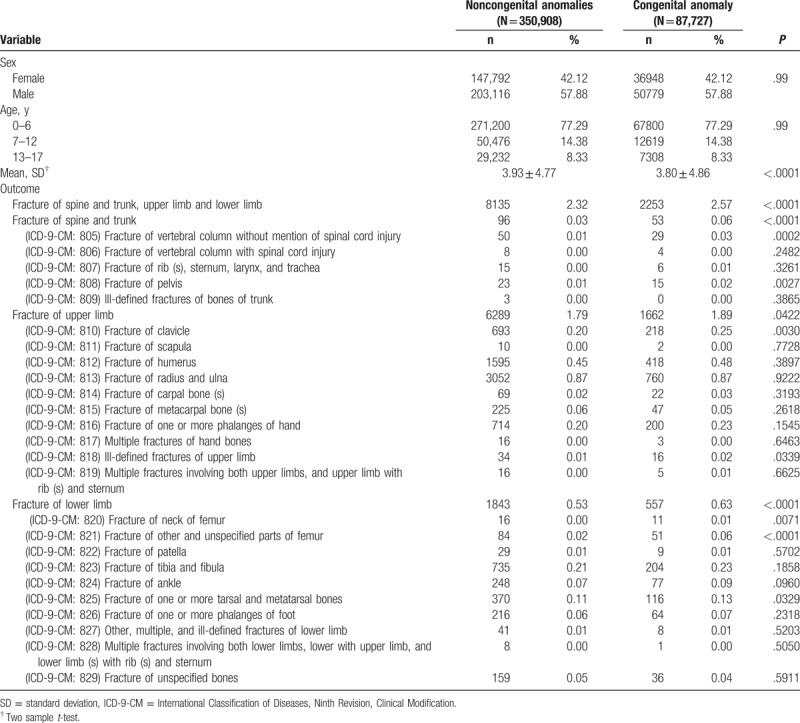
Demographic characteristics in patients with congenital anomaly and without congenital anomalies diseases.

Male patients represented 57.88% of each group. The age distribution was the same in both groups. Patients 0–6 years of age comprised 77.29% of the study population; patients 7–12 years of age comprised 14.38%; and those 13 to 17 years of age comprised 8.33% of the study population. The mean age of the control group was 3.93 ± 4.77 years, and the mean age of patients with congenital anomalies was 3.80 ± 4.86 years. Both groups suffered from fractures of the spine, trunk, upper, and lower limbs. In patients without congenital anomalies, 2.32% suffered fractures, whereas 2.57% of patients with congenital anomalies suffered fractures. Patients with congenital anomalies had a significantly higher statistical probability to be affected by a fracture of spine, trunk, upper, or lower limbs.

We also detailed the fracture location. Significant differences (*P* < .05) between the 2 groups were noted for the following fracture locations: vertebral column excluding spinal cord injury, pelvis, clavicle, ill-defined upper limb, neck of femur, other unspecified parts of femur, and 1 or more tarsal and metatarsal bones. We used the Cox model regression analysis to determine HRs and 95% confidence intervals for fractures associated with congenital anomalies and covariates (Table 2). The hazard ratio of congenital anomalies associated with all fractures was 1.11 (95% confidence interval = 1.06–1.17, *P* < .001). To be more exact, fracture of the spine and trunk showed a 2.22 hazard ratio (95% confidence interval = 1.59–3.10, *P* < .001), fracture of the upper limbs showed 1.06 hazard ratio (95% confidence interval = 1.01–1.12, *P* = .0321), and fracture of the lower limbs showed 1.22 hazard ratio (95% confidence interval = 1.11–1.34, *P* < .001).

**Table 2:**
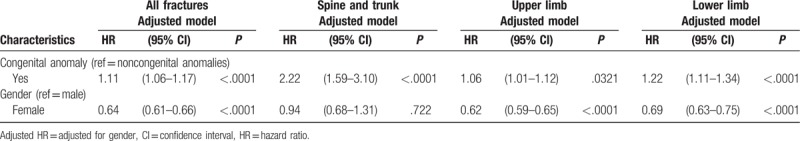
Cox model measured hazard ratio and 95% confidence intervals of fracture of spine and trunk, upper limb, and lower limb associated with congenital anomaly diseases and covariates.

Table 3 shows the incidence rates, hazard ratios, and confidence intervals for fractures, stratified by sex, for patients with congenital anomalies. When compared to control, male and female patients in the congenital anomalies group had a significantly higher HR (1.11), with a 95% confidence interval of 1.02–1.21 for female patients and 1.05–1.17 for male patients.

**Table 3:**
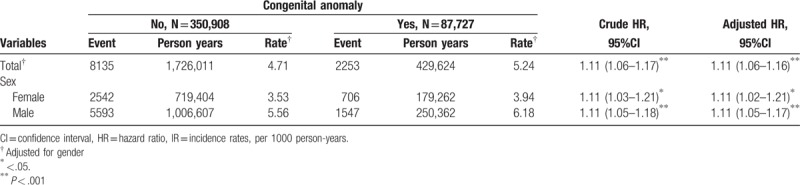
Incidence rates, hazard ratio, and confidence intervals of fracture of spine and trunk, upper limb, and lower limb for with and without congenital anomaly patients in the stratification of gender.

Table 4 shows the average age of fracture in patients with congenital anomalies, classified by fracture location. Fracture of the vertebral column, without mention of spinal cord injury, occurred at the highest mean age of all fractures. Multiple fractures, involving upper limbs, and upper limb with rib(s) and sternum, showed the lowest mean age of occurrence.

**Table 4:**
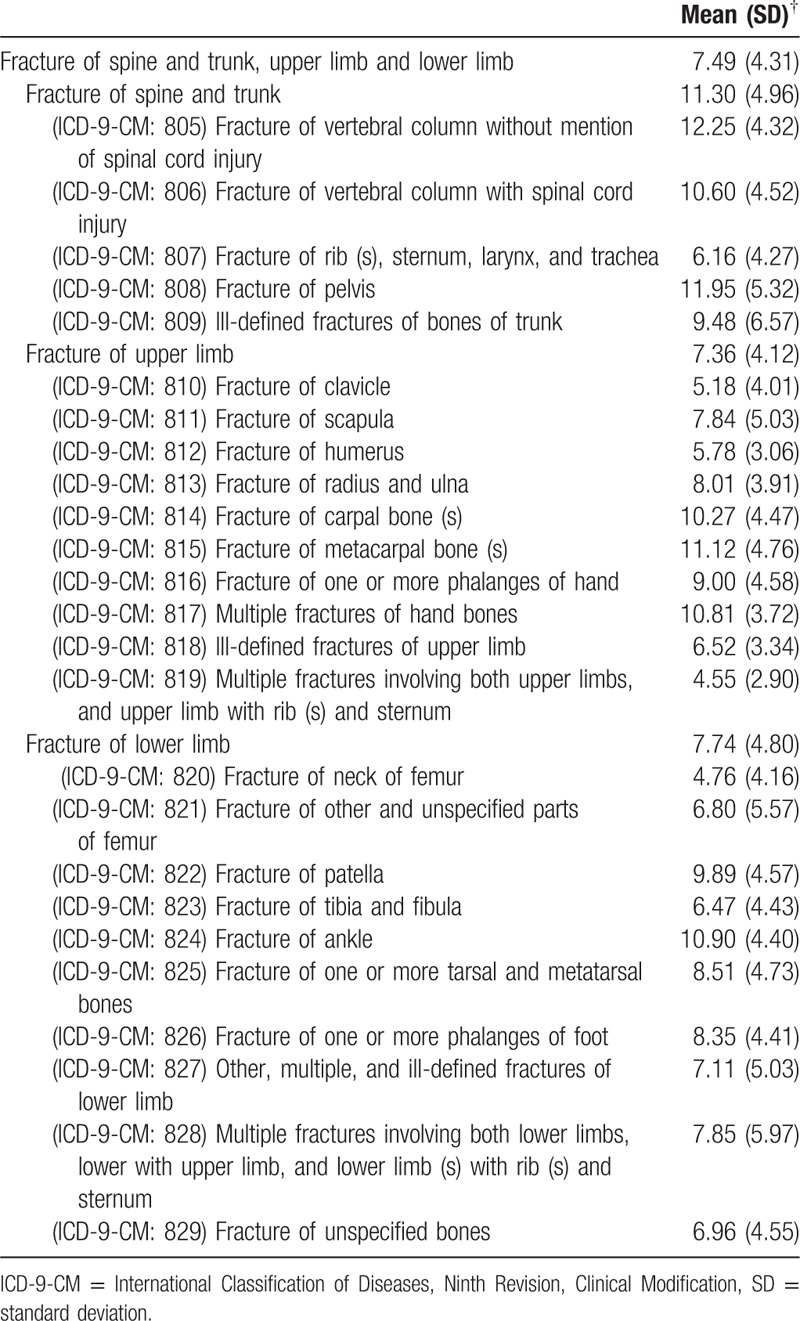
Average age in fracture of spine and trunk, upper limb and lower limb.

## 4 Discussion

In our study, the incidence rates of fracture were higher in patients with congenital anomalies. These results correspond with the TCM principle that “the Kidney governs the quality of bones.”^[6]^

Congenital anomalies are significant medical and public health issues, and in developing countries, constitute one of the major causes of infant mortality.^[7]^ Birth defects severity ranges from minor and correctable, to mental and physical disabilities, and fatal anomalies. Older maternal age is a known risk factor for chromosomal anomalies.^[3,8]^ Additional risk factors include fetal factors, such as plurality and sex; parental factors, such as ethnicity, socio-economic status; lifestyle (including tobacco and alcohol consumption, drug use, and medication during pregnancy); body weight; congenital disease; and environmental exposure.^[7,9–15]^ The occurrence rates of congenital anomalies in Taiwan are as follows: nervous system is 0.67%; eyes and face is 1.86%; cardiovascular 1.47%; digestive is 0.62%; urogenital is 0.71%; musculoskeletal is 2.05%; respiratory systems is 0.07%; and chromosomes is 0.79%.^[3]^

Twenty-five percent of childhood fractures are the result of accidents and injuries.^[16]^ In our study, fractures were more common among teen-aged boys than among teen-aged girls. These included fractures of the vertebra, skull, rib, ankle, pelvis, and patella. Other fractures were prevalent during early childhood, such as greenstick, humerus, radius/ulna, carpus, and foot.^[16]^ In some cases, nutritional factors are related to an increased fracture risk in children.^[17]^ In addition, chronic illness may be related to poor bone health, resulting in low bone minerals accrual and increased resorption. Pediatric disorders associated with low bone mineral density, and significant fracture risk in adolescence and adulthood, include celiac disease, type I diabetes mellitus, and cystic fibrosis.^[18]^ In our study, we excluded patients with traumatic fractures of spine, trunk, upper and lower limbs. We designed this study to test the TCM theory^[19–21]^ that “the Kidney governs the bones,” in which kidney malfunctions would manifest as congenital anomalies, arising from poor bone health quality, and eventually result in a fracture.^[6]^

In Western medicine, several clinical studies have identified an association between congenital anomalies and fracture. For example, patients with spina bifida (myelomeningocele) experience fractures of the lower extremities, with an estimated incidence ranging from 11% to 30%.^[22]^ In 1987, Rosenstein et al^[23]^ described that bone density of the upper and lower extremities in patients with spina bifida is related to the neurologic level, and ambulatory status. In 1998, Quan et al reported that bone mineral density of the distal radius in patients with spina bifida was 1 to 2 standard deviation units below that in the normal population. They concluded that patients with lower bone mineral densities are at risk of suffering pathologic fractures.^[24]^ In 2010, Szalay and Cheema^[25]^ reported that children with spina bifida are at risk of having low bone density, which contributes to fractures. Recently, Kafadar et al^[26]^ concluded that lower bone mineral density identified in patients with spina bifida is associated with osteoporosis, rather than nutritional and hormonal factors.

Other congenital anomalies are associated with fracture and low bone mineral density. Patients with Loeys–Dietz syndrome, an autosomal dominant connective tissue disorder marked by aortic aneurysms, have a high risk of fracture, and a high incidence of low bone mineral density.^[27]^ Children with Alagille syndrome are reported to be at risk of pathologic fractures, which commonly appear at an early age, and are specifically observed in the lower extremity of long bones.^[28]^ Pathologic fractures of the tibia are associated with Charcot–Marie–Tooth disease, and pathological fractures of the mandible in a pediatric patient are associated with congenital insensitivity to pain with anhidrosis.^[29,30]^ Patients with the Sjögren–Larsson syndrome experience pathological femoral neck fractures. Spontaneous fractures occur in patients with the Prune–Belly syndrome, a rare disorder characterized by partial or complete absence of the abdominal muscles, failure of testes to descend, and/or urinary tract malformations.^[31,32]^ These previous reports prompted us to investigate the relationship between congenital anomalies and fracture.

A trend showing increased fracture incidence in patients with congenital anomalies was observed in our research. In this study, patients with congenital anomalies have poor quality of bone, which can be explained by both traditional Chinese medicine and modern Western medicine concepts. In TCM theory, the kidney dominates the foundation of an individual's innateness and contributes to the origin of congenital constitution, whereas congenital anomalies arising from kidney malfunction, results in unhealthy bones, and eventual fracture. Similarly, in Western medicine, chronic illnesses are associated with poor bone health, due to lower bone mineral accrual and increased resorption. Our study design excluded patients with congenital anomalies and with previously diagnosed fracture and patients who were diagnosed with more than 2 fractures. Also, excluded in our study were patients with congenital anomalies who suffered from trauma, which may cause fractures. Moreover, we identified the specific fracture locations and the patients mean age. In conclusion, our study is more reliable because of the concentration of the statistics based only on patients with congenital anomalies without previous history of bone problems.

According to TCM, there are 5 important viscera: liver, heart, spleen, lung, and kidney. Each organ has its own corresponding internal organs. In Huang Di Nei Jing (Yellow Emperor's Canon of Internal Classic, Inner Canon of the Yellow Emperor), the Kidney govern the bones, and healthy bones give the body stabilization, and prevent fractures.^[33,34]^ Our findings support this principle, which is also compatible with concepts from modern Western medicine. Regular follow-up for bone qualities in patients with congenital anomalies is recommended. When specific findings are observed, a complete orthopedic examination and advanced testing should be performed. Further studies should evaluate if treatments are necessary and whether correcting congenital diseases in patients can prevent future fracture.

Our study has strength when compared to previous studies. The database is heterogeneous, based on nationwide data, and data were obtained from different hospitals, medical centers, and clinics. The study population is larger, and longer in scope, than previous studies.

To the best of our knowledge, this is the first research study to support the following TCM theory: “the kidney governs the bones, and healthy bones give the body stabilization and prevent fracture events.” The results revealed that compared to controls, the incidence of fractures of the spine, trunk, and upper and lower limbs is higher in patients with congenital anomalies.
